# History and prospects of intracytoplasmic sperm injection (ICSI) and the development of golden hamster ICSI embryos

**DOI:** 10.1002/rmb2.12410

**Published:** 2021-08-26

**Authors:** Nami Morishita, Masanori Ochi, Toshitaka Horiuchi

**Affiliations:** ^1^ Institute for Advanced Reproductive Medicine Ochi Yume Clinic Nagoya Nagoya Japan; ^2^ IVF Lab Ochi Yume Clinic Nagoya Nagoya Japan; ^3^ Emeritus Professor of Prefectural University of Hiroshima Hiroshima Japan

**Keywords:** development, embryo, golden hamster, intracytoplasmic sperm injection

## Abstract

**Background:**

Golden (Syrian) hamsters have many advantages for the study of reproductive biology and developmental biology, including a consistent estrous cycle, a stable superovulation response, and a short gestation period. However, there are serious difficulties in doing in vitro manipulations of hamster embryos, because they are very sensitive to various environmental factors. Thus, biotechnological researches of hamster embryos should be performed with high‐level skills of embryo manipulations.

**Methods:**

The authors summarized the history of hamster intracytoplasmic sperm injection (ICSI) and introduced key points for hamster ICSI, which were found in our previous studies on the production of embryos by ICSI and offspring by embryo transfer.

**Main findings:**

The key points for hamster ICSI were in vitro manipulations under the light‐controlled environment, injection of acrosome‐less sperm heads into oocytes as soon as possible before spontaneous oocyte activation occurs, and determination of the optimal culture conditions.

**Conclusion:**

To our knowledge, there are no available reports on production of offspring from ICSI embryos in hamsters except our reports. Moreover, success rates of hamster ICSI remain very low. For the purpose of spreading hamster ICSI, it is necessary to make further researches to improve manipulation techniques and to resolve experimental problems.

## 1 HISTORY OF HAMSTER INTRACYTOPLASMIC SPERM INJECTION

The first success of in vitro production of fertilized oocytes with the intracytoplasmic sperm injection (ICSI) was performed with oocytes from the golden hamster (hereafter referred to as hamster; Table 1).^1^ Later, pronuclear formation was observed after injecting testicular spermatozoa, and caput or caudal epididymal spermatozoa into hamster oocytes.^2^ Other studies applied ICSI techniques to clarify the timing of sperm nuclear chromatin decondensation, pronuclear formation, and DNA synthesis.^3^, ^4^ In addition, interspecies differences in the stability of mammalian sperm nuclei were clearly shown in the comparative experiments in which spermatozoa from the hamster, human, mouse, chinchilla, rat, and bull were injected into hamster oocytes.^5^

**TABLE 1:** History of the development of the intracytoplasmic sperm injection (ICSI) and the round spermatid injection (ROSI) into golden hamster oocytes

Year	Author	ICSI/ROSI	Sperm source	Sperm treatment	Results
1976	Uehara and Yanagimachi^1^	c‐ICSI	Cauda epididymal	Homogenized	PN formation; the first report of fertilization by ICSI in mammals
1977	Uehara and Yanagimachi^2^	c‐ICSI	Testicular, caput, and cauda epididymal	Homogenized, sonicated (some samples)	PN formation
1987	Perreault et al^3^	c‐ICSI	Testicular, caput, and cauda epididymal	Sonicated, DTT‐treated	Timing of SP nuclear decondensation and PN formation
1987	Naish et al^4^	c‐ICSI	Caput and cauda epididymal	Sonicated, DTT‐treated	Decondensation, PN formation, and DNA synthesis
1988	Perreault et al^5^	c‐ICSI	Cauda epididymal	Sonicated	Time course of decondensation of SP nuclei: 45–60 min
1991	Yanagida et al^6^	c‐ICSI	Testicular, caput, and cauda epididymal	Sonicated & heated at 60–125°C for 20–120 min	PN formation
					Thermostability of sperm nuclei
1992	Katayose et al^7^	c‐ICSI	Cauda epididymal	Freeze‐dried	PN formation; stored 12 months at 4°C
1993	Ogura et al^12^	e‐ROSI	Spermatid	–	69.4% 2‐cell
1993	Ogura and Yanagimachi^13^	e‐ROSI	Round spermatid	–	73.4% 2‐cell
2002	Yamauchi et al^8^	p‐ICSI	Cauda epididymal	Frozen‐thawed & piezo‐pulse separation	48.6% (121/249) morulae
					19.1% (9/47) live pups
2004	Haigo et al^10^	p‐ROSI	Round spermatid	–	51.3% (59/115) morulae
					21.7% (25/115) blastocysts
					5.3% (3/57) live pups
2008	Kanatsu‐Shinohara et al^19^	p‐ROSI	Derived from male germ stem cells	–	3.8% (4/104) morulae/blastocysts
2011	Muneto and Horiuchi^11^	p‐ICSI	Cauda epididymal	Freeze‐dried & piezo‐pulse separation	62.2% (23/37) morulae
					16.2% (6/37) blastocysts
					13.0% (3/23) live pups
2014	Pan et al^18^	p‐ICSI	Cauda epididymal	Piezo‐pulse separation	84.7% 2‐cell
2019	Morishita et al^17^	p‐ICSI	Cauda epididymal	Frozen‐thawed & sonicated	78.4% (69/88) morulae
					42.0% (37/88) blastocysts
					9.3% (4/43) live pups

Abbreviations: c‐ICSI, conventional‐ICSI; DTT, dithiothreitol; e‐ROSI, electrofusion‐ROSI; p‐ICSI, piezo‐ICSI; PN, pronuclear; p‐ROSI, piezo‐ROSI; SP, sperm.

After several basic research studies by the Perreault group in 1987–88,^3^, ^4^, ^5^ Yanagida et al. (1991)^6^ reported that the thermostability of hamster sperm nuclei was related to disulfide cross‐linking of sperm protamines. Then, Katayose et al. (1992)^7^ reported that dehydrated sperm nuclei could develop into individual pronuclei. However, at that time, a culture system for hamster zygotes had not been established. Thus, it was not possible to study in vitro embryo development beyond the 2‐cell stage or the transfer of ICSI embryos into hosts to produce offspring. In 2002, twenty‐five years after the first hamster ICSI, Yamauchi et al^8^ reported the first birth of offspring from hamster ICSI embryos which were produced according to the mouse piezo‐ICSI method of Kimura and Yanagimachi (1995).^9^ In that report, Yamauchi et al. described the separation of heads from tails in hamster spermatozoa with piezo‐pulses and the injection of acrosome‐less sperm heads into oocytes. In 2004, in our laboratory, Haigo et al. produced the first birth of offspring from round spermatid injection (ROSI) into oocytes by using Yamauchi method.^10^ Then, in 2011, Muneto and Horiuchi^11^ reported the first birth of offspring from hamster ICSI embryos produced by injecting freeze‐dried spermatozoa into oocytes. The success of the ROSI method was supported by the pioneering efforts of Ogura et al. (2003),^12^ and Ogura and Yanagimachi (2003),^13^ who developed the electrofusion ROSI approach.

The methods of Yamauchi et al.,^8^ Haigo et al.,^10^ and Muneto and Horiuchi^11^ required high‐level skills of micromanipulation for quick separation of relatively tighter association between heads and tails in hamster spermatozoa by using piezo‐pulses. Moreover, there was a time limit for the procedure of hamster ICSI, because hamster oocytes had the tendency to undergo parthenogenetic activation during ICSI process.^14^, ^15^, ^16^ Therefore, we applied the sonication treatment of the separation of sperm heads from tails in order to simplify the hamster ICSI technique. Especially, we found that the use of Tris‐HCl buffer with EGTA was valid for conserving chromosomal integrity of sperm head during the sonication treatment. Consequently, this new technique allowed us to produce embryos more efficiently leading to the achievement of birth of offspring by rapid injection of the sperm nuclei.^17^

Despite these efforts for technical improvements, hamster ICSI is still suffering from low efficiency of in vitro early development of embryos and birth of offspring after embryo transfer. For instance, Pan et al. (2014)^18^ reported that hamster ICSI embryos severely stopped at the 2‐cell stage (2‐cell block). Although hamster offspring could be produced from ROSI embryos as described previously,^10^ the microinjection of hamster round spermatids generated from cultured male germline stem cells had problems that these ROSI embryos stopped early development by the morula stages.^19^ Thus, it is very difficult to manipulate hamster spermatozoa, oocytes, and embryos in vitro. In order to overcome these difficulties, it is necessary to refine the procedures of hamster ICSI. In this review, we introduced key points for a successful ICSI and proposed the utilization of ICSI embryos in hamsters.

## 2 OVERCOMING THE CHALLENGE OF PRODUCING LIVE OFFSPRING WITH HAMSTER ICSI

### 2.1 Keys to the success of hamster ICSI

**FIGURE 1:**
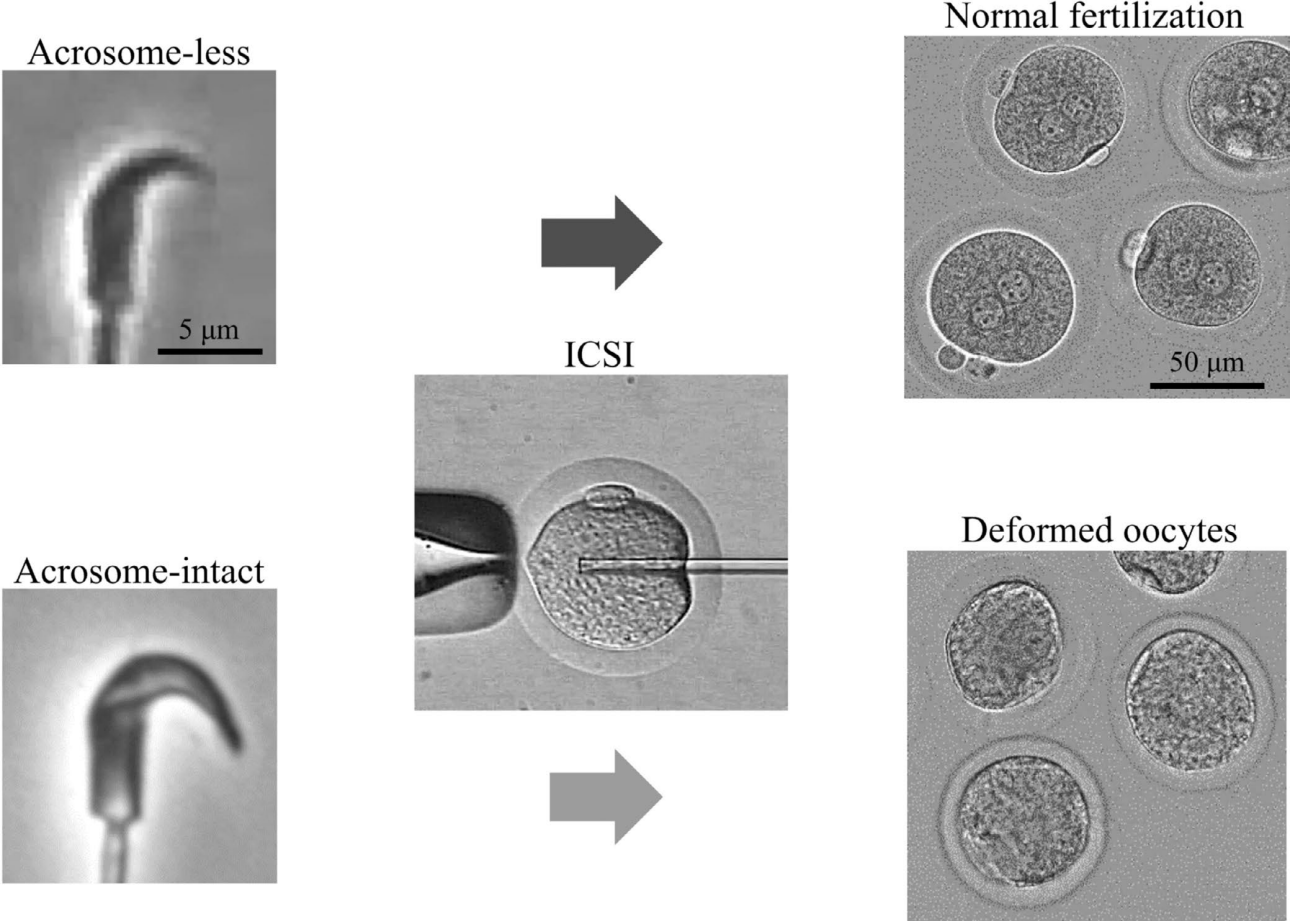
Hamster oocytes should be fertilized by acrosome‐less sperm injection, not acrosome‐intact. Hamster acrosome can be observed by using phase‐contrast microscope (acrosome‐less: top left, a sperm head with dark tip region, acrosome‐intact: bottom left, a sperm head with white tip region). Acrosome‐less sperm was fertilized normally (top), but acrosome‐intact sperm was deformed oocytes (bottom). Scale bars are 5 µm, in the sperm photograph, and 50 µm, in the oocytes photograph

As mentioned in section 1, only four studies,^8^, ^10^, ^11^, ^17^ all performed in our laboratory, have achieved the in vitro development of injected oocytes beyond the 2‐cell stage, into morulae and blastocysts. In addition, we successfully transferred hamster ICSI embryos into recipients and produced offspring. In our experience, three keys to the success of hamster ICSI were (1) manipulating oocytes/zygotes in a dark room and performing ICSI under a microscope with a red‐light source, (2) injecting sperm heads without acrosomes (Figure 1), and (3) performing ICSI as soon as possible, before spontaneous oocyte activation occurs.

#### 2.1.1 Avoid light damage

During in vitro manipulations of oocytes or embryos, ambient light affects their subsequent in vitro development.^20^, ^21^ We handled hamster oocytes and zygotes under a microscope light covered with a piece of red cellophane in a dark room. When embryos were manipulated under typical room lighting conditions, none of the cleaved embryos developed beyond the 2‐cell stage.^8^ Most researchers that work with mouse embryos do not believe that light can damage embryos during in vitro manipulations, because when mouse embryos are manipulated in vitro under light conditions, they develop normally to blastocysts. Thus, some researchers might think that light only affects golden hamster embryos under unusual, special, or exceptional conditions. If you think that, please see the study by Takenaka et al. (2007).^22^ Indeed, when hamster or mouse zygotes were exposed to 1200 lx of cool white fluorescent light for 15 min, 97% of hamster zygotes underwent the first cleavage, but did not divide further. In contrast, 100% of mouse zygotes exposed to the same light developed into blastocysts. However, the incidence of apoptotic cells was high in mouse blastocysts in the light‐exposed group, compared with the light‐controlled group. The light exposure induced the production of reactive oxygen species (ROS) in zygotes, which led to a developmental block in hamster embryos and a high incidence of apoptosis in mouse blastocysts.^22^ Nakayama et al. (1994) showed that exposure to visible light significantly increased ROS in hamster embryos.^23^ Much more ROS was produced in hamster zygotes than in mouse zygotes. Therefore, an important point in performing ICSI is to manipulate both oocytes and embryos in a dark room, under a microscope with a red‐light source. In our studies, all in vitro handling of hamster oocytes and embryos, particularly unfertilized eggs and pronuclear embryos, was strictly conducted under light‐controlled conditions.^8^, ^10^, ^11^, ^17^

#### 2.1.2 Remove acrosomes from spermatozoa

The second key to the success of hamster ICSI is to remove the acrosomes from spermatozoa.^8^ When hamster oocytes are injected with acrosome‐intact spermatozoa, they grow deformed and die. Conversely, when hamster oocytes are injected with acrosome‐less heads, they survive and develop into live offspring (Figure 1). The acrosome is a cap‐like structure that covers the anterior portion of the sperm head. This structure contains hydrolyzing enzymes^24^ that are detrimental to the cytoplasm of hamster oocytes. Yamauchi et al. (2002)^8^ removed the acrosomes from sperm heads by freeze‐thawing the spermatozoa. Then, the sperm heads without acrosomes were separated from the tail by applying piezo‐electric pulses.

In human ICSI, high fertilization and pregnancy rates were achieved by injecting the entire motile spermatozoon, after immobilization.^25^ Unlike hamster oocytes, human oocytes can survive the injection of acrosome‐intact spermatozoa, because human sperm acrosomes are very small, and the small amount of hydrolyzing enzymes cannot compromise oocytes. However, when four or more human spermatozoa were injected into mouse oocytes, the multiple human spermatozoa produced vacuole‐like structures within the ooplasm.^26^

#### 2.1.3 Perform ICSI immediately after oocyte retrieval

The third important point for a successful hamster ICSI is to perform the ICSI as soon as possible, before the onset of spontaneous oocyte activation. It is well known that, after oocytes are collected from the oviducts and cumulus cells are removed, unfertilized hamster oocytes readily undergo spontaneous activation.^14^, ^15^, ^16^ Thus, during the ICSI, hamster oocytes may initiate activation. Within 1 h after starting the ICSI, a cytoplasmic protrusion appears on the surface of the oocyte, and this is followed by the excursion of the second polar body. Therefore, it is important to perform ICSI as soon as possible, before spontaneous activation occurs. In mice, the MII stage is strongly maintained in oocytes, and the spontaneous activation rate increases as the post‐ovulatory oocyte age increases. When hamster oocytes were collected from oviducts at 15 and 17 h after the hCG injection and cultured for 6 h, the oocyte activation rate increased markedly at 17 h (unpublished results). The spontaneous activation of hamster oocytes in vitro is oocyte age‐related.^18^

Yamauchi et al. (2002)^8^ and Haigo et al. (2004)^10^ removed acrosomes from sperm heads by freezing and thawing. Then, they separated the heads from the tails with piezo‐electric pulses. In frozen‐thawed hamster spermatozoa, the heads are relatively tightly bound to the tails, and it takes time to separate them with piezo‐electric pulses. Thus, the efficiency of the piezo‐ICSI method was very low for producing fertilized eggs. As mentioned above, hamster oocytes are prone to spontaneous activation. Therefore, the ICSI must be performed immediately after oocyte retrieval. In the early days of developing hamster ICSI,^2^ sonication was introduced for sperm separation. This method is very simple, and it is more efficient than the piezo‐electric method for performing ICSI. However, some studies found that sonicating spermatozoa caused chromosome fragmentation, similar to freeze‐drying spermatozoa.^27^ We found that this damage was not caused by the sonication, but by the activation of DNase by Ca^2+^, which was present in the treatment solution. Sperm sonication with a Tris‐HCl solution supplemented with EGTA significantly improved the efficiency of hamster ICSI (by 2‐ to 3‐fold). In addition, the developmental rate was improved, due to the preservation of chromosome integrity.^17^

### 2.2 In vitro hamster ICSI embryo cultures

Hamster embryos are developmentally blocked in vitro at both the 2‐ and 4‐cell stages, due to their high susceptibility to a variety of factors.^28^, ^29^, ^30^, ^31^ The development of culture media for in vivo‐fertilized hamster embryos can be attributed largely to the work of Bavister and colleagues.^32^, ^33^, ^34^, ^35^, ^36^, ^37^ They described modifications to the culture medium that enabled in vivo‐fertilized 1‐cell hamster embryos to develop beyond the 2‐cell stage to blastocysts. These modifications included removing glucose, phosphate, and pyruvate, reducing lactate and adding glutamine. Currently, hamster embryo culture medium‐3 (HECM‐3) is the basic medium for culturing zygotes in vitro. Later, the basic HECM was improved by supplementing it with amino acids and vitamins. The HECM series is chemically defined, and instead of serum albumin, it contains polyvinyl alcohol (PVA). Although the medium improved the efficiency of culturing hamster embryos, it remained difficult to induce in vitro‐fertilized (IVF) hamster embryos to develop into blastocysts,^34^ much less ICSI embryos.

In our laboratory, two types of culture media were designed for in vitro cultures of hamster ICSI zygotes. The first was TCM199 supplemented with 5% fetal bovine serum (FBS), 5 mM taurine, and 25 µM EDTA (M199TE, Table 2). We used this medium for culturing ICSI zygotes up to the 2‐cell stage. We supplemented TCM199 with 5% FBS, because in an interspecies in vitro fertilization study, an analysis of human sperm chromosomes showed that this medium produced the highest rate of chromosome appearance in zona‐free hamster oocytes.^38^ Furthermore, we added taurine and EDTA to reduce environmental stress. However, M199TE contains glucose and phosphate, which cause a 2‐cell block in hamster embryos. Thus, in this medium, it is impossible to culture embryos consistently beyond the 2‐cell stage.

**TABLE 2:** Components of M199TE and modified hamster embryo culture medium‐9 (HECM‐9)

M199TE		
Component	Concentration	Amount in 10 ml
M199^a^	–	9.5 ml
FBS	5%	0.5 ml
Taurine	5.0 mM	6.3 mg
EDTA·2Na·2H_2_O^b^	25.0 µM	25 µl

Modified HECM9		
Component	mM	mg/100 ml, unless otherwise indicated
NaCl	113.8	665.0
KCl	3.0	22.4
CaCl_2_·6H_2_O	1.9	27.9
MgCl_2_·6H_2_O	0.5	10.2
NaHCO_3_	25.0	210.0
Phenol red	–	1.0
Na‐lactate (60% syrup)	4.5	0.06 ml
Glutamine	0.2	2.9
NEAA (×100)^c^	1×	10 µl/ml
Alanine	0.1	
Asparagine	0.1	
Aspartic acid	0.1	
Glutamic acid	0.1	
Glycine	0.1	
Proline	0.1	
Serine	0.1	
Taurine	5.0	0.63 mg/ml
Pantothenate (×100)^d^	0.003	10 µl/ml
Human serum albumin (HSA)	0.5 mg/ml	

Note

*Modified HECM9*: The pH was adjusted by adding 0.45 µl/ml of 1 M HCl.

The modified HECM‐9 was stored at 4°C for no more than 1 week prior to an experiment.

^a^ Medium 199 with 2.2 g/L NaHCO_3_ + 25 mM HEPES (GIBCO, #12340‐030).

^b^ EDTA stock:18.6 mg/5 ml Milli‐Q water.

^c^ NEAA: MEM non‐essential amino acids (GIBCO, #11130).

^d^ Pantothenate (×100): 1.4 mg Ca‐Pantothenate in 10 ml.

The second culture medium we designed was a modification of HECM‐9. Original HECM‐9 is a simple composition medium (HECM‐3) with 9 amino acids (0.01 mM each of asparagine, aspartic acid, cysteine, glutamic acid, glycine, histidine, lysine, proline, and serine), 0.5 mM taurine, 3 µM pantothenate and 0.1 mg/ml PVA.^37^ With this medium, Yamauchi et al. (2002)^8^ showed that ICSI 2‐cell embryos could develop into morulae, but not into blastocysts. Therefore, we needed to improve the original HECM‐9 to induce ICSI 2‐cell embryos to develop into blastocysts. We modified the amino acid content (0.1 mM each of alanine, asparagine, aspartic acid, glutamic acid, glycine, proline, serine), and we substituted the 0.5 mM taurine and PVA with 5 mM taurine and 0.5 mg/ml human serum albumin (HSA) (Table 2). With this modified medium (mHECM‐9), in vivo‐fertilized 2‐cell hamster embryos developed into blastocysts at high rates (>90%). Moreover, in this medium, ICSI 2‐cell hamster embryos developed to morulae and blastocysts, and after an embryo transfer, they produced offspring.^10^, ^11^, ^17^

**FIGURE 2:**
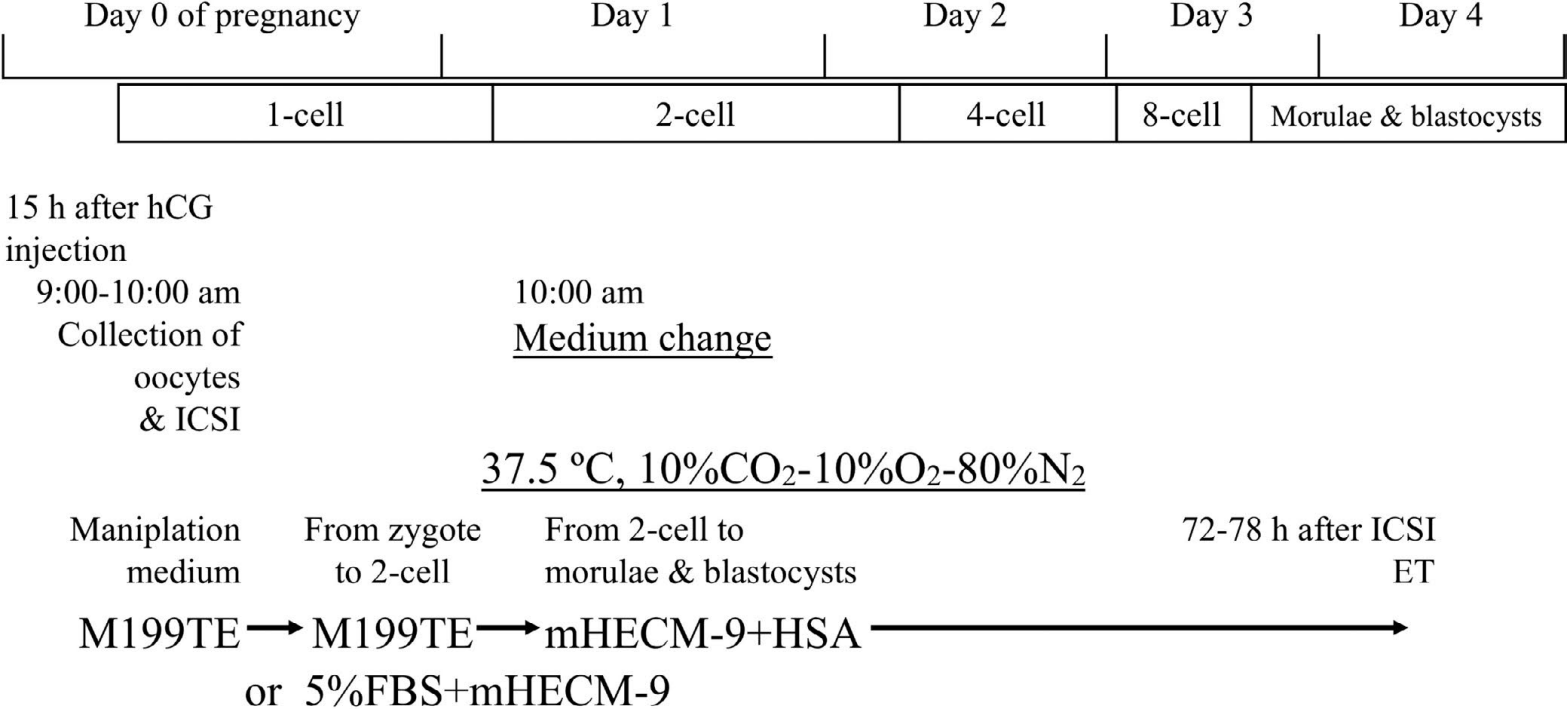
Time schedule for culturing hamster intracytoplasmic sperm injection (ICSI) embryos. At 15 h after the hCG injection, hamster oocytes were collected, and ICSI was performed in M199TE. Then, hamster embryos were cultured in M199TE, or mHECM‐9 supplemented with 5% FBS, and incubated at 37.5℃, with 10% CO_2_, 10% O_2_, and 80% N_2_. At 24 h after ICSI, the embryos were transferred to mHECM‐9 supplemented with 0.5 mg/ml HSA. At about 72–78 h after ICSI, hamster ICSI embryos developed into morulae and blastocysts. All medium changes were performed at 10:00 a.m.

The time schedule we used for culturing ICSI embryos is shown in Figure 2. At 15 h after the donor hamster was injected with hCG, oocytes were collected and placed in M199TE for ICSI. After ICSI, zygotes were cultured in M199TE for 24 h. Recently, for this step, instead of M199TE, we have cultured zygotes in mHECM‐9 supplemented with 5% FBS. At 24 h after ICSI, the embryos were transferred to mHECM‐9 supplemented with 0.5 mg/ml HSA. About 72–78 h after ICSI, hamster ICSI embryos developed into morulae and blastocysts. The medium changes are very important for continuous development of hamster embryos.

The gas‐phase condition for hamster embryo incubations was maintained at 10% CO_2_, 10% O_2_, and 80% N_2_. This gas‐phase condition is essential for hamster embryo cultures, because it influences pH adjustments and ROS production in the culture environment. Indeed, in vitro cultures of 2‐cell and 8‐cell hamster embryos developed into morulae and blastocysts at significantly higher rates in 10% CO_2_ than in 5% CO_2_ and at higher rates in 10% O_2_ than in 20% O_2_.^35^, ^39^ On the other hand, 5% O_2_ is often used for in vitro cultures of hamster pronuclear embryos.^40^, ^41^ The intracellular pH of hamster embryos is 7.24. Therefore, the CO_2_ concentration for in vitro cultures should be higher than 5%.^40^, ^42^

## 3 DEVELOPMENT AND APPLICATION OF HAMSTER ICSI EMBRYOS

Hamster ICSI is a useful model for improving ICSI technology. Using our in vitro culture system, most of in vivo‐fertilized zygotes developed into blastocysts (about 80%). However, in vitro‐fertilized zygotes (such as with ICSI) could not developed into blastocysts at such a high rate (about 40%).^17^ Reduced developmental competence of hamster ICSI‐fertilized zygotes into blastocysts is dependent on the fertilization process by ICSI. This result suggested that the ICSI method has some problems that affect embryos before the pronuclear stage, for example, oocyte activation by sperm injection and the following male and female pronuclear formation.

Currently, there are two types of human embryo culture media: sequential medium^43^ and single medium.^44^ Although there is much debate about the superiority of these, they are used differently, depending on laboratory efficiency and patient background. A continuous culture system for hamster embryo production has not been established, because we lack medium with a clear composition that can generate a high rate of development from pronuclear embryos. When a single culture medium for hamster embryos is invented that anyone can reproduce, it will definitely represent a breakthrough. A stress‐free hamster embryo culturing method could lead to many new applications.

**FIGURE 3:**
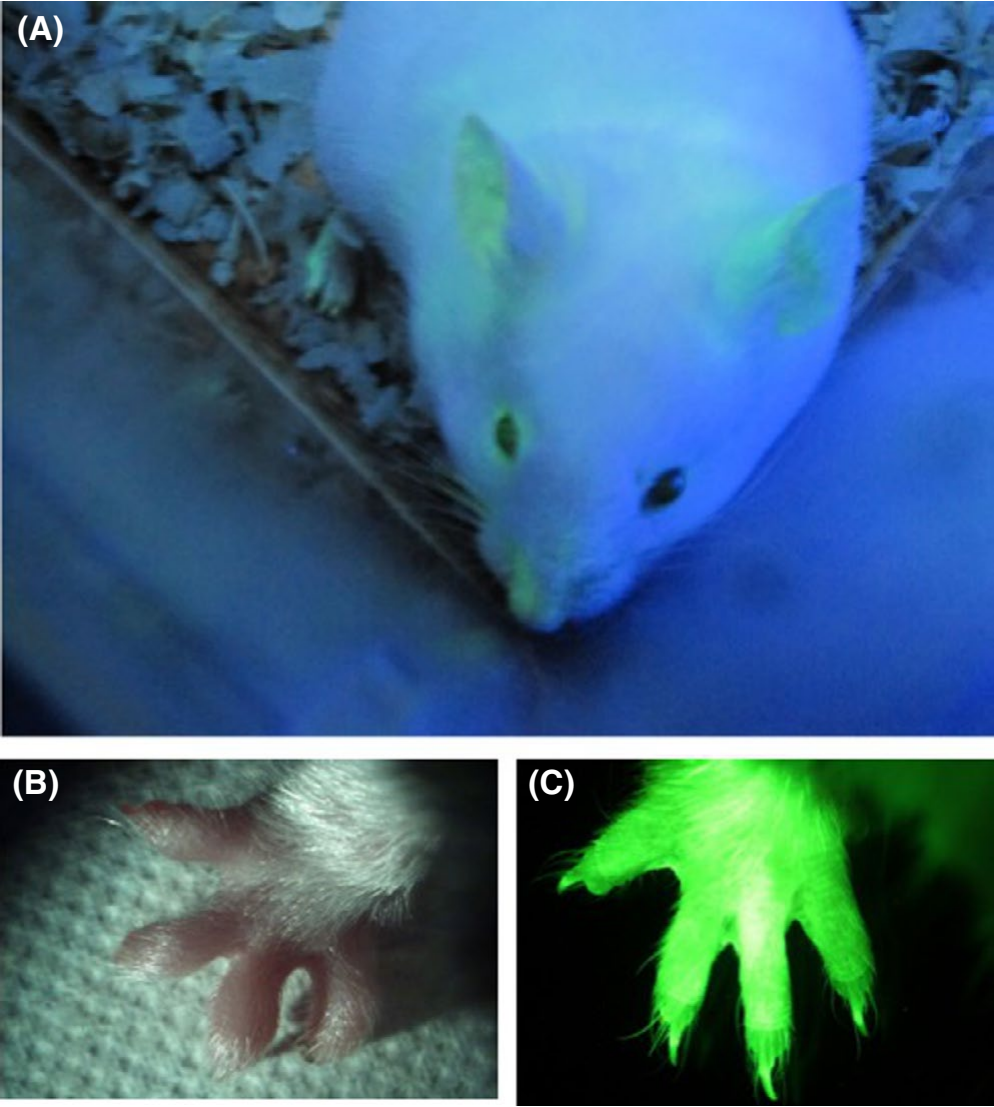
A transgenic hamster that expresses green fluorescent protein (GFP), produced with ICSI of freeze‐dried sperm heads mixed with pCX‐EGFP DNA fragments. (A) In blue light, green tinting in the ears and nose indicate that GFP is expressed in transgenic hamster skin. GFP expression is easy to detect in hamster fingers, shown (B) in a bright field and (C) in UV light [Colour figure can be viewed at wileyonlinelibrary.com]

Recently, new genome editing technology has been used to produce knockout (KO) hamsters without in vitro embryo development. The KO hamsters have provided new information, different from that gained previously in mouse models.^45^, ^46^ Many transgenic hamsters can be produced with this technique, without the need for embryo manipulation in vitro. However, in vitro culture systems combined with IVF and ICSI will be needed to investigate issues related to embryo and fetal development. Exactly, there were many restrictions to produce transgenic hamsters due to the developmental block, because it is not easy to culture hamster embryos. In our laboratory, we produced one green fluorescent protein (GFP)‐expressing ICSI hamster offspring by mixing freeze‐dried sperm heads with pCX‐EGFP DNA fragments before performing ICSI (Figure 3, doctoral thesis of T. Muneto).

The low production efficiency of hamster ICSI requires improvement. It is well known that, in most mammals, in vitro‐fertilized embryos develop more slowly than in vivo‐fertilized embryos. Indeed, the developmental speed of hamster ICSI embryos was about half a day slower than that of in vivo‐fertilized embryos. In hamster embryo transfers, synchronization of the donor embryos and recipient females was important for embryo implantation and development in vivo,^29^, ^34^, ^47^ because the implantation time for embryo transfer is very limited. One solution to this problem is the cryopreservation of hamster embryos. However, few studies have investigated the storage of frozen hamster embryos.^48^, ^49^ It is essential to establish a reliable technique for the cryopreservation of hamster embryos to facilitate the widespread use of transgenic hamsters in the future.

## 4 CONCLUSION

The golden hamster is a useful animal for research on IVF/ICSI and embryo development, for example, due to evaluate the relationship between fertilization events and the subsequent embryo development. In addition, the use of hamsters in biomedical research might provide new knowledge that is difficult to gain in a mouse model, for example, they have recently attracted attention as a small animal model for SARS‐CoV‐2 infections and countermeasure development.^50^ In the hamster, the fertilized oocytes can be stably obtained by ICSI, and the timing of sperm injection is clear. Although it was not a high success rate, we showed that hamster ICSI embryos could develop into blastocysts in vitro and we could produce live offspring after ICSI embryo transfer. At the present, the research on the interaction between ICSI and embryo development will be performed followed by the time course of fertilization events. Improvements in the ICSI technique for over 26 years have continued to overcome developmental blocks and facilitated the production of live pups. Here, we highlighted the most important steps for achieving a successful ICSI: (1) manipulate the eggs under a light‐controlled environment, (2) inject sperm heads without acrosomes, (3) perform ICSI as soon as possible after collection, and (4) select the optimal culture system: use M199TE for culturing embryos up to the 2‐cell stage, and use mHECM‐9 for culturing from 2‐cells to blastocysts. Additionally, the incubation environment should be maintained at 10% CO_2_, 10% O_2_, and 80% N_2_. The rate of ICSI embryo development to the blastocyst stage and the rate of producing pups remain relatively low. Therefore, more improvements are needed to make ICSI practical for widespread use.

## CONFLICT OF INTEREST

Nami Morishita, Masanori Ochi, and Toshitaka Horiuchi declare no conflict of interest.

## HUMAN/ANIMAL RIGHTS

This review article included no patients, and thus, it did not require approval from an Ethics Committee.

## HUMAN RIGHTS STATEMENT AND INFORMED CONSENT

This study did not contain any human materials.
